# Erythema Multiforme Major Associated With Community-Acquired Pneumonia: Lessons From a Case Report

**DOI:** 10.3389/fped.2021.698261

**Published:** 2021-07-29

**Authors:** Xiaomei Fan, Yong Luo, Jieluan Lu, Jinji Xu, Qing Chen, Huijuan Guo, Ping Jin

**Affiliations:** ^1^Shenzhen Baoan Women's and Children's Hospital, Jinan University, Shenzhen, China; ^2^College of Pharmacy, Jinan University, Guangzhou, China

**Keywords:** azithromycin, erythema multiforme major, human leukocyte antigen, adverse drug reactions, children

## Abstract

**Background:** Erythema multiforme (EM) is an acute immune-mediated inflammatory mucinous skin disorder. The etiology of pediatric EM involves infections, medications, autoimmune diseases, and genetic factors.

**Case Report:** An 8-year-old girl with *Mycoplasma pneumoniae* (MP) associated community-acquired pneumonia developed erythema target-like symptoms 1 week after azithromycin administration. The erythema quickly spread throughout the body involving the oral and ocular mucous membranes, the trunk, and the extremities, and eventually developed into erythema multiform major (EMM). Through drug withdrawal and specific treatment including systemic corticosteroids and supportive care, her clinical symptoms were improved. After 31 days, most of the mucocutaneous symptoms were relieved, except pigmentation. Human leukocyte antigen (HLA) gene sequencing was performed and 20 HLA genotypes were identified. The patient follow-up lasted for 18 months. Rashes appeared on her trunk when receiving azithromycin orally after discharge and then disappeared after azithromycin withdrawal.

**Conclusions:** Pediatric EM is a rare disease and recognition of its etiology is important for EM management. In this case, azithromycin and HLA-DQB1^*^03:01 genotype may contribute to EMM.

**Lesson:** For drug-induced EM, rapid identification and withdrawal of the causative drugs is critical. Re-exposure to the same drug or exposure to drugs with similar chemical structures should also be avoided. Patient education and rational use of medicines are essential for pediatric patients.

## Introduction

Erythema multiforme (EM) is an acute immune-mediated inflammatory mucinous skin condition characterized by urticarial papules, red spots, plaques, bilateral iris's vesicles target, or limb distribution iris (1). This condition is often confused with more serious conditions such as Stevens-Johnson syndrome (SJS) and toxic epidermal necrosis (TEN). Bastrop-Garin et al. were first to report EM based on its morbid physiology, etiology, and clinical processes (2, 3). EM is clinically characterized by mucocutaneous involvement and classified into erythema multiforme minor and erythema multiforme major (EMM; severe mucosal damage ≥2 mucous coat) (4–6). It is associated with the onset of early systemic symptoms including fever and mucosal involvement with cutaneous lesions appearing after a few days. Clinical incidences of EM are <1% which mainly affect children and young adults.

However, the pathobiological mechanisms underlying the onset of EM have not been fully elucidated. Infections, medications, immunizations, and autoimmune diseases are regarded as etiological agents for EM. Herpes simplex virus (HSV) is the primary cause of EM, accounting for about 70% of the cases, while *Mycoplasma pneumoniae* (MP) is considered the second most common etiological agent for EM, especially in children (7, 8). In addition, numerous drugs are associated with EM, especially non-steroidal anti-inflammatory drugs, antibiotics [amoxicillin (9), norfloxacin (10), cephalothin (11), trimethoprim-sulfamethoxazole (12)], and antiepileptics. Barbiturates, phenothiazines, statins, and TNF-α inhibitors, among others are also associated with EM onset (13, 14).

Some severe cutaneous adverse reactions (SCARs) encoded by human leucocyte antigen (HLA) have attracted clinical attention (15). Specific HLA gene alleles have been reported to contribute to the increasingly severe skin reactions such as SJS/TEN and EMM, in particular, severe cutaneous adverse drug reactions (cADRs) (16, 17). For instance, HLA-B^*^15:02, HLA-B^*^15:11, and HLA-B^*^46:01 are potential risk factors for carbamazepine-induced SJS/TEN in Asian population (18, 19). Among Thai patients, HLA-B^*^46:01 and HLA-A^*^02:07 were found to contribute to the pathogenesis of phenytoin and lamotrigine-related cutaneous reactions, respectively (20, 21). HLA-B^*^58:1 is associated to both drug reactions with eosinophilia and systemic symptoms (DRESS) and epidermal necrolysis caused by allopurinol (22). The HLA-DQB1^*^03:01 genotype is a common marker in herpes-associated EM (HAEM) (23).

In this case report, a pediatric patient with severe *Mycoplasma pneumonia* who later developed EMM was described. HLA genotypes of the child and her parents were detected to evaluate the association between EM and HLA alleles.

## Case Report

**Figure 1:**
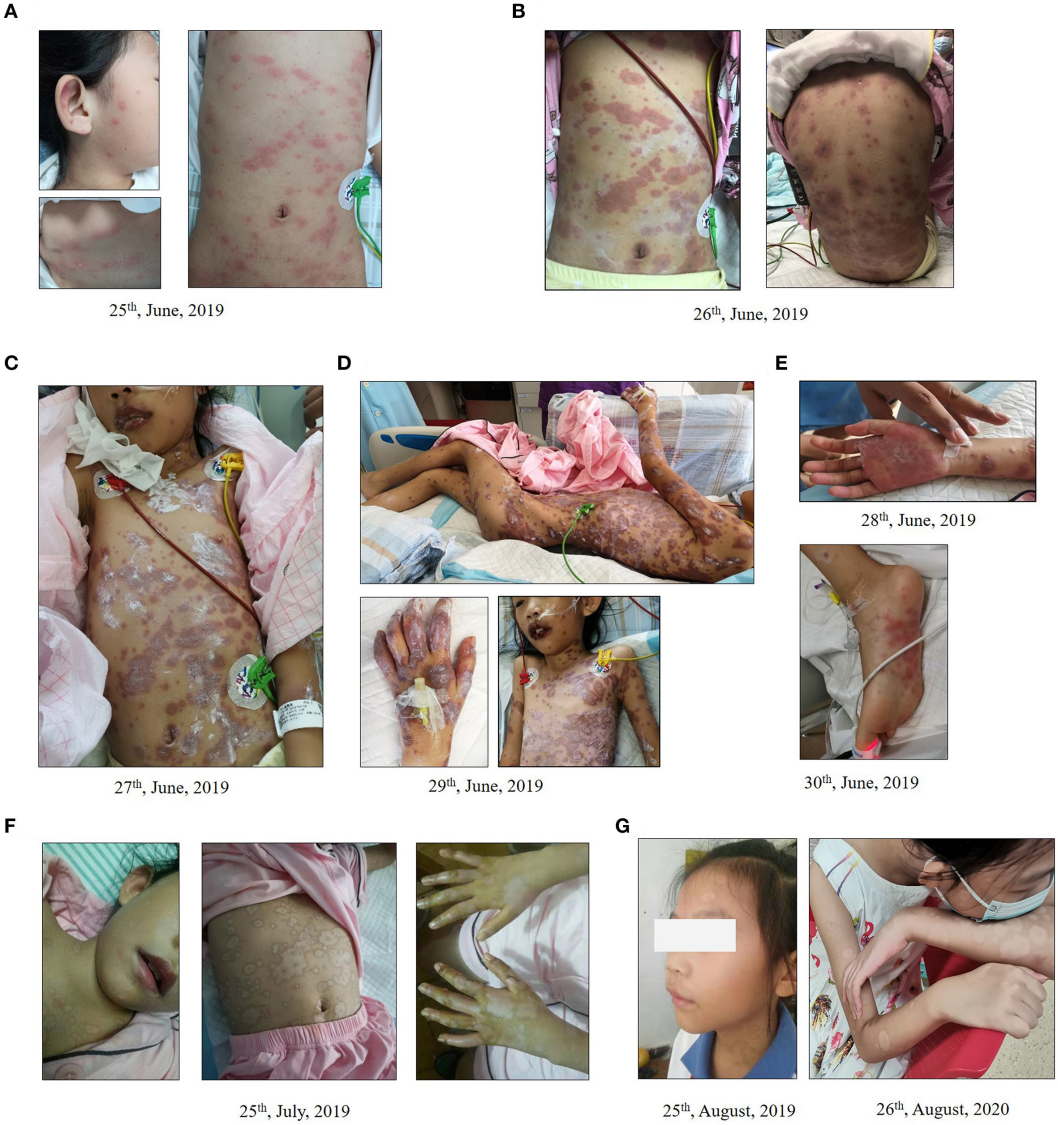
Dynamic changes of erythema multiforme major in an 8-year-old Chinese girl. **(A,B)** Initiation **(A)** and progression **(B)** of erythema multiforme with painful blistering spread in the chest, back, and limbs of the child. **(C–E)** Cutaneous erythema was further exacerbated **(C,D)** and new rashes occurred on her hands and feet **(E)**. **(F)** Local ulcerations on lips and oral mucosa and systemic rash improved after 4 weeks of treatment. Rashes on the lower extremities and abdomen were scab-free. **(G)** Pigmentation persisted on her arms and shoulders where erythema multiforme had occurred during the follow-up visit after discharge.

An 8-year-old Asiatic girl from south China with acute bronchopneumonia was admitted to the hospital on June 24th, 2019. She had experienced an 8-day unprovoked fever with axillary temperature peaking at 39.5°C, a 6-day dry cough and pharyngeal congestion. Prior to admission, she had been administered with azithromycin (200 mg, qd) and ibuprofen (7 mg/kg, q6h as recommended) for 3 days without clinical remission. The chest X-ray test demonstrated lower left lobe pneumonia. A day after admission, no rales was heard with a stethoscope. Sporadic miliary rashes with raised target (three concentric rings) were observed on her trunk (Figure 1A). Pulmonary infections with fever, listlessness, high respiratory rate (32/min), and high heart rate (128/min) persisted. One or more ocular symptoms, including swollen eyelids, conjunctival hyperemia, increased secretion, and photophobia accompanied by erosive lesions of lips developed. Blood tests showed elevated C-reactive protein (CRP) (77.2 mg/L [normal: <5 mg/L]) and procalcitonin (PCT) (0.12 ng/mL [normal: <0.05 ng/mL]) levels, increased erythrocyte sedimentation rate (ESR) (59 mm/h [normal: 0–34 mm/h]), and high neutrophil percentage (81.4% [normal: 43–75%]). The serological test for HSV-1 and HSV-2 IgM were negative. Other blood parameters such as white blood cell counts were normal. Two days after admission, progressive erythema with painful blistering and raised typical target lesions with three-concentric rings spread on her chest, back, and limbs (Figure 1B), indicating severe erythema multiforme. Electrocardiogram examination revealed sinus tachycardia. Severe MP evidenced by abnormally elevated MP antibodies (IgG [>300 AU/ml [normal: <24.0]) and IgM (5.01[normal: <0.9 S/CO]), left-lower-lobe pneumonia, pleural effusion, multiple infectious lesions in lungs, and dyspnea (shortness of breath) was subsequently diagnosed. Then, she was transferred to the pediatric intensive care unit (PICU). Azithromycin (10 mg/kg/d) was continuously intravenously administered for 5 days. Gamma globulin (γ-globulin) (1.0 g/kg/d for 2 days) and a steroid infusion (methylprednisolone 120 mg/d for 2 days, 200 mg/d for 3 days, then 40 mg/d for 4 days) were followed by sequential oral prednisone.

After 3 days of admission, erythema developed progressively and further invaded her feet and perineum, and erosion on the lips and oral mucosa was observed (Figure 1C). The color of existing lesions changed to dark-red hue from red. Meanwhile, bilateral acute purulent conjunctivitis had developed for 3 days, and levofloxacin eye drops were used for treatment. General body temperature levels returned to normal following 3-day treatment and pulmonary infection symptoms were subsequently alleviated. Pulmonary auscultation revealed minor moist rales. However, cutaneous erythema was further exacerbated (Figure 1D) and new rashes occurred on her hands and feet on the 6th day after admission (Figure 1E). Given the possibility of drug-induced EMM, highly-associated medications including azithromycin and ibuprofen were withdrawn on the 7th day of treatment and all unnecessary medications were discontinued. Erythromycin eye ointment and calamine lotion were used for lip, mucosa and skin care. Local ulcerations on lips, oral mucosa and systemic rashes were gradually improved over the following weeks of treatment, with some of them scabbing and desquamating. On the 30th day of therapy, rashes on the upper and lower extremities and abdomen were completely scab-free (Figure 1F). Complete blood count (CBC), electrolyte, liver and kidney functions, myocardial enzyme, CRP, PCT, and urine routines tests were in the normal ranges, and the patient was discharged.

The child was clinically monitored for 18 months through follow-up, and no erythema multiforme was reported. At the sites of erythema multiforme, pigmentation persisted on her arms and shoulders (Figure 1G). Allergic rhinitis and cough were diagnosed at 18 months after discharge. Azithromycin was used at home by self-medication in the absence of medical supervision. Rashes appeared on her trunk at 10 h after receiving azithromycin (orally) and then diminished following azithromycin withdrawn and oral administration of antihistamine (loratadine) according to doctor's advice.

## Family History and Pedigree

**Figure 2:**
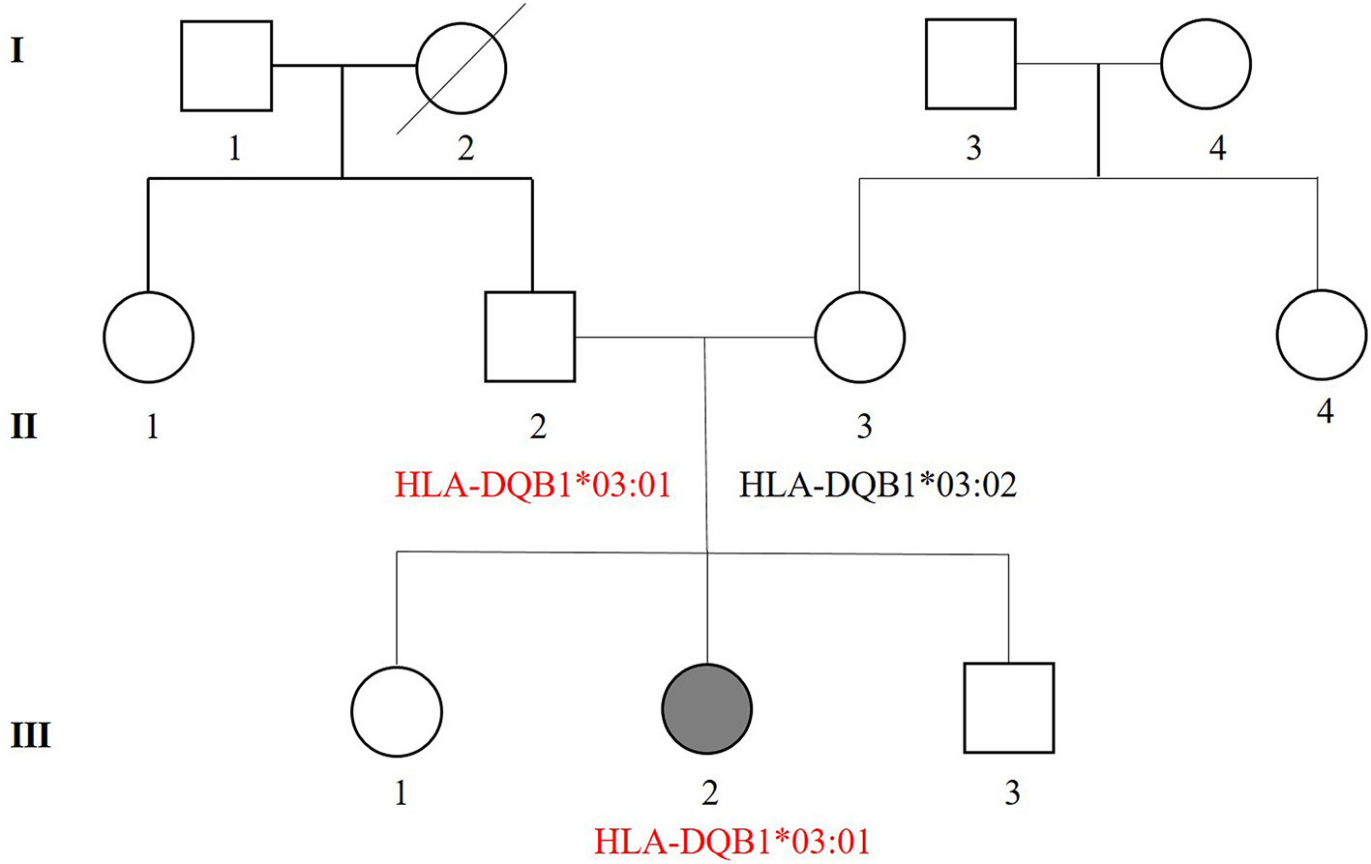
Pedigree of the three-generation (I–III) patient's family. Squares denote males while circles denotes females. Shadow symbols indicate affected individuals, while open symbols represent unaffected individuals. Diagonal lines are used to show that a person is died. Information regarding HLA genotypes associated with EM is also included in the pedigree. II-2 without a history of EM shows the same haplotypes (HLA-DQB1*03:01) as the patient with EMM (III-2).

The pedigree chart of the patient's family is presented in Figure 2. The patient had a history of alopecia areata and developed red skin when sweating. Her mother had erythema eruption caused by recurrent herpes virus on her trunk twice in her childhood, and also suffered urticarial papules, and cutaneous pruritus, and xerodermia. Her father had not suffered any dermatosis. The grandfather had an early bald. It remains unknown whether members of generation I in the pedigree chart had suffered any skin disorders.

### Immunogenetic Sequencing

After signing an informed consent, peripheral blood samples (2 mL) were collected from the child and her parents for HLA gene sequencing to determine the association between HLA alleles and EMM. After genomic DNA extracted from peripheral blood cells, the HLA alleles including HLA class I (A, B, C), class II (DQA1, DQB1, DPA1, DPB1, DRB1, DRB3, DRB4, DRB5), and HLA-G were genotyped by polymerase chain reaction and Sanger sequencing-based typing (PCR-SSBT) with Applied Biosystems®3730 DNA Analyzers. The uTYPE®HLA Sequencing Software was used to perform the analysis. The sequencing results of the family are shown in Table 1. Twenty HLA genotypes were identified for the patient such as HLA-A ^*^02:03, HLA-A ^*^02:07, HLA-B ^*^15:25, and HLA-B ^*^46:01. Some HLA genotypes are reported to be highly-associated with cutaneous adverse reactions including EMM and SJS/TEN (Table 2). The HLA genotypes overlapped between the patient and her parents were marked and mapped into a genetic pedigree (Figure 2).

**Table 1:** HLA genotypes of the patient and her parents.

**Haplotypes**	**Genotypes**					
	**Patient**		**Patient's mother**		**Patient's father**	
HLA-A	*02:03	*02:07	*02:07	*11:01	*02:03	*31:01
HLA-B	*15:25	*46:01	*15:01	*46:01	*15:25	*51:01
HLA-C	*01:02	*03:04	*01:02	*04:01	*03:04	*14:02
HLA-DQA1	*03:02	*06:01	*03:01	*03:02	*01:02	*06:01
HLA-DQB1	*03:01	*03:03	*03:02	*03:03	*06:02	*03:01
HLA-DPA1	*01:03	*02:02	*01:03	*02:02	*01:03	*01:03
HLA-DPB1	*04:02	*05:01	*02:01	*05:01	*02:01	*04:02
HLA-DRB1	*09:01	*12:02	*04:06	*09:01	*12:02	*15:01
HLA-DRB3	*03:01	/	/	/	*03:01	/
HLA-DRB4	*01:03	/	*01:03	*01:03	/	/
HLA-DRB5	/	/	/	/	*01:01	/
HLA-G	*01:01	*01:01	*01:01	*01:01	*01:01	*01:03

*HLA alleles marked in blue are those that have been correlated with cutaneous adverse reactions*.

**Table 2:** Relevant genotypes-based pharmacogenomics relationships between HLA alleles and cutaneous adverse reactions.

**Genotypes**	**SCARs**	**cADRs**
HLA-A*02:07	TEN in Japanese population (24)	Zonisamide-induced SJS/TEN in Japanese population (18); Lamotrigine-induced cADRs in Thai patients (21); Clarithromycin-induced cADRs in Chinese Patients (25); Tetanus-induced exanthematous drug eruptions in the Han Chinese population (16)
HLA-B*46:01	-	Carbamazepine (CBZ) -induced SJS/TEN in Chinese Population (19); Phenytoin (PHT) -induced cutaneous ADR in Thai patients (20);
HLA-C*01:02	-	Methazolamide-induced SJS/TEN in Han Chinese (26);
HLA-C*03:04	-	Co-trimoxazole (CTX) -induced SJS/TEN and DRESS in Thai (15); Cold Medicine (CM)-induced SJS/TEN with Severe Ocular Complications (SOC) in the Korean population (27);
HLA-DQB1*03:01	Herpes-associated EM in Caucasian (23)	-
HLA-DQB1*03:03	-	Carbamazepine (CBZ)-induced SJS/TEN among Han individuals from northeastern China (28)
HLA-DRB1*09:01	-	Allopurinol-induced hypersensitivity in Asian (PharmGKB)
HLA-DRB1*12:02	-	Co-trimoxazole induced cADRs in Thai patients (29);
		Acetaminophen-induced SJS/TEN with severe ocular complications (SOC) in Japanese individuals (30)

*HLA genotypes presented in this table were chosen from the HLA sequencing results of our patient*.

## Discussion

EM is an acute mucocutaneous hypersensitivity reaction triggered by various etiological factors (31, 32). Timely recognition and treatment of EM is a major challenge. Due to the similarities and overlaps among EM, SJS and TEN, there are some controversies over the precise classification of these skin disorders (33). It is crucial to distinguish between EM and SJS/TEN and choose different treatment approaches. These various clinical entities are defined by the morphology of the individual lesions and their pattern of distribution (34). SJS or SJS/TEN mainly presents macules or flat atypical target lesions and rapidly evolves as a blistering disorder of the skin and mucosal surfaces (1). Conversely, EM are characterized by predominantly acral target lesions but frequently truncal target lesions in children. Typical target lesions which are raised are only found in patients with EM (34). Additionally, MP-induced rash and mucositis (MIRM) emerged as an entity in 2015, which mostly affects mucous membranes and has minimal or even absent cutaneous involvement (35, 36). MIRM has a milder disease course than EMM and SJS/TEN (8). In our case report, raised typical target lesions, involvement of the trunk and extremities, extensive mucosal involvement (≥2 sites, oral and ocular mucous membranes) and systemic symptoms presented are consistent with the diagnostic criteria of EMM except for the body surface area (BSA) of skin lesions. Although we acknowledge that the extensive body surface area affected in our patient would be more consistent with SJS classification according to the consensus classification of severe blistering skin reactions (34), based on the characteristics reported above and the consultation of a pediatric dermatologist from our hospital, a final diagnosis of EMM was eventually made.

Identification of the etiologic agent for EM is important for its management. The most common causes include infections such as herpes simplex and mycoplasmal infection, medications, and autoimmune conditions (31). HSV-associated EM usually presents classic raised target lesions without mucosal involvement. In this case, the HSV IgM in serum was negative and mucosal involvement was observed, ruling out HSV-associated EM secondary to HSV infection. On the 6th day after admission, MP infection was gradually improved after treatment, however, mucocutaneous eruptions was further exacerbated and new rashes occurred on her hands and feet. It suggests that EM was unlikely induced by MP. Drugs are the second most frequent causes of EM (31). In pediatric patients, antibiotics are responsible for the greatest proportion of adverse reactions in EM (57%) and SJS/TEN (30%) hospitalizations (37). Some of the drugs which the patient had taken before the onset of EM can induce cutaneous adverse drug reactions according to the post-marketing information (13). Azithromycin and ibuprofen were highly suspected to induce EM in this case. However, ibuprofen had been discontinued for 5 days when EM appeared. The patient had taken ibuprofen several times in her childhood due to the fever, but didn't develop any cADRs. It suggests that the causative agent is probably azithromycin rather than ibuprofen. Azithromycin, a potent and well-tolerated semisynthetic macrolide from erythromycin, is the most prescribed antibiotic in China. Macrolide antibiotics have been reported to induce severe hypersensitivity related cutaneous reactions, which are rare ADRs (38–40). Pediatric cases have been found to develop SJS associated with azithromycin and reactivation of HSV infections (41). Additionally, SARS-CoV-2-related EM was also reported in adult and pediatric COVID-19 cases and the persons receiving mRNA COVID-19 vaccines (42–44).

Increased hypersensitive reactions to certain drugs may be correlated to specific HLA antigens (45). Studies have revealed the association between HLA alleles and EM, giving more insight in EM pathogenesis (45, 46). HLA gene sequencing was performed in this case and twenty HLA alleles were identified. All the HLA genotypes in the patient are hereditary and those correlated with SCARs and cADRs are summarized in Table 2. HLA-C^*^03:04, HLA-A^*^02:07, and HLA-DQB1^*^03:03 are associated with cADRs such as SJS/TEN and drug reactions with eosinophilia and systemic symptoms (DRESS) in different populations (15, 18, 21, 28). HLA-DQB1^*^03:01 is the most frequent allele in EM patients and highly correlated with herpes-associated EM in a case-control study of the Caucasian population (23). HLA-DQB1^*^03:02 and HLA-DQB1^*^04:02 alleles have been reported to be associated with recurrent HSV-induced EM involving mucous membranes (47). Based on the sequencing results and the association studies between HLA genotypes and cutaneous adverse reactions, we postulate that HLA-DQB1^*^03:01 plays a more important role in EMM than other detected alleles (23, 47). There was a genetic resemblance that was associated with the three-generation (I-III) haplotypes in the patient's family. Notably, HLA-DQB1^*^03:02 was noticed based on HLA genotypic differences between the patient and her mother. The patient had no sign of HLA-DQB1^*^03:02 but her mother had. The history of erythema eruption caused by recurrent herpes virus was confirmed in her mother during a follow-up visit.

EM therapy depends on etiology, disease severity, clinical manifestations, as well as the development of EM (1). Clinical management of EM includes symptomatic treatment with topical corticosteroids and antihistamines and treatment for the underlying etiology. Systemic corticosteroids have been regularly used to treat acute EM for decades (48, 49), although their roles are unknown. In highly suspicious drug-induced EM cases, rapid identification and withdrawal of the causative drugs are critical to avoid immune reactions worsening. Furthermore, re-exposure to the same drug or exposure to drugs with similar chemical structures which may have a potential of cross-reactivity should be avoided. It is essential to strengthen drug patient education and promote rational use of medicines for pediatric patients.

## Conclusions

Pediatric EM is an acute, immune-mediated condition affecting the skin and mucosal surfaces in children. An unexpected erythema in an 8-year-old Chinese girl broke out after azithromycin treatment for MP infection, which later developed to EMM. Clinical symptoms of EMM were improved through suspected drug withdrawal and specific treatments. Drugs and genetic factors may have contributed to the EMM-like symptoms. This is the first case report on an Asiatic azithromycin-induced EMM child positive for HLA-DQB1^*^03:01. However, causality between EMM and azithromycin needs to be further elucidated. Whether HLA-DQB^*^03:01 plays a role in drug-induced EMM remains unknown due to limited information from a single case. Study involving more pediatric patients will be performed to explore the pathological mechanisms and potential genetic characteristics of drug-induced EMM.

## Data Availability Statement

The original contributions presented in the study are included in the article/supplementary materials, further inquiries can be directed to the corresponding authors.

## Ethics Statement

The studies involving human participants were reviewed and approved by Baoan Women's and Children's Health Hospital ethics committee. Written informed consent to participate in this study was provided by the participants' legal guardian/next of kin. Written informed consent was obtained from the minor(s)' legal guardian/next of kin for the publication of any potentially identifiable images or data included in this article.

## Author Contributions

YL, JX, and PJ contributed to patient's care and clinical data. XF, YL, JL, QC, and HG analyzed the data and wrote the first draft of the manuscript. XF, HG, and PJ contributed to the final version of the manuscript. All authors read and approved the final manuscript.

## Conflict of Interest

The authors declare that the research was conducted in the absence of any commercial or financial relationships that could be construed as a potential conflict of interest.

## Publisher's Note

All claims expressed in this article are solely those of the authors and do not necessarily represent those of their affiliated organizations, or those of the publisher, the editors and the reviewers. Any product that may be evaluated in this article, or claim that may be made by its manufacturer, is not guaranteed or endorsed by the publisher.

## Abbreviations

cADRs: cutaneous adverse drug reaction
CBC: complete blood count
CRP: C-reactive protein
DRESS: drug reactions with eosinophilia and systemic symptoms
EM: Erythema multiforme
EMM: erythema multiforme major
ESR: erythrocyte sedimentation rate
HAEM: herpes-associated EM
HLA: human leukocyte antigen
HSV: Herpes simplex virus
MP: *Mycoplasma pneumonia*
PCT: procalcitonin
PICU: pediatric intensive care unit
SCARs: severe cutaneous adverse reactions
SJS: Stevens-Johnson syndrome
TEN: toxic epidermal necrosis
WBC: white blood cell.
